# Epidemiology of Primary Urethral Cancer: Insights from Four European Countries with a Focus on Poland

**DOI:** 10.3390/cancers18020290

**Published:** 2026-01-17

**Authors:** Iwona Wnętrzak, Urszula Wojciechowska, Joanna A. Didkowska, Jakub Dobruch, Mateusz Czajkowski, Roman Sosnowski

**Affiliations:** 1 Department of Urology, Centre of Postgraduate Medical Education, Independent Public Hospital of Professor W. Orlowski, 00-416 Warsaw, Poland; 2 Polish National Cancer Registry, Maria Sklodowska-Curie National Research Institute of Oncology, 02-781 Warsaw, Poland; 3 Department of Epidemiology and Cancer Prevention, Maria Sklodowska-Curie National Research Institute of Oncology, 02-781 Warsaw, Poland; 4 Department of Urology, Medical University of Gdańsk, Mariana Smoluchowskiego 17 Street, 80-214 Gdańsk, Poland; drmatczajkowski@gmail.com; 5 Department of Urology and Oncological Urology, MSWiA Hospital, Warmian-Masurian Cancer Center, 10-228 Olsztyn, Poland

**Keywords:** primary urethral cancer, Poland, Europe, incidence, mortality, rare urogenital tumors

## Abstract

**Background/Purpose**: Primary urethral cancer is a rare malignancy, accounting for less than 1% of all urogenital cancers. Current epidemiological data from Europe are scarce and outdated. Therefore, the analyzes and comparison of the incidence and mortality of PUC in selected European countries, with particular focus on Poland, based on the most recent available registry data, were performed. **Methods**: Our study is based on country-level data and is descriptive in nature. Incidence data for PUC were obtained from the national cancer registries of Poland, Latvia, Slovenia, and Hungary. Mortality data were sourced from the WHO Mortality Database. Age-standardized incidence rates were calculated for two time intervals (2000–2009 and 2010–2019). Age-standardized mortality rates for individuals aged ≥45 years were calculated using the European Standard Population (ESP2013). Trends in incidence and mortality in Poland were analyzed using a five-year moving average. **Results**: The highest incidence of PUC was observed in Hungary, while Poland showed the lowest incidence. Latvia had the highest ASMRs for both sexes, whereas Poland and Greece reported the lowest mortality rates. Despite slight annual fluctuations, the overall PUC mortality rate in Poland has remained stable. Our study is limited by the relatively short analyzed period (2000–2021), restricted availability of C68.0 incidence data from national cancer registries, and incomplete mortality data in the WHO mortality database. **Conclusions**: This first contemporary comparative analysis of PUC epidemiology in Europe highlights the rarity of this malignancy and the limited data availability. Based on the knowledge drawn from the literature presented in the article on the impact of centralization on the increase in overall survival and the decrease in mortality in rare cancers, the authors believe that centralization of care can improve PUC patient outcomes.

## Simple Summary

Primary urethral cancer is a very rare cancer of the urinary tract, and current information on how often it occurs and causes death in Europe is limited and often outdated. This makes it difficult to understand the scale of the problem and to improve patient care. In this study, the authors analyzed the most recent population-based cancer registry and mortality data from selected European countries, with a particular focus on Poland. The aim was to compare differences in disease occurrence and death rates between countries and over time. The results confirm that primary urethral cancer is uncommon, affects men more often than women, and shows clear variation across Europe. By identifying gaps in data availability and highlighting the potential role of specialized treatment centers, this study provides valuable insights for researchers and clinicians working to improve outcomes in rare cancers.

## 1. Introduction

Primary urethral cancer (PUC) is classified by the European Union as a rare condition, with an incidence rate of less than one case per 2000 individuals [1]. Across the 28 European Union member states, PUC exhibits a prevalence of 3986 cases, with 1504 new cases documented annually, constituting less than 1% of all urogenital malignancies [2]. Additionally, PUC occurs three times more frequently in males than in females [2]. Among the three main categories of rare urogenital cancers, primary urethral cancers extend beyond penile and upper urinary tract cancers.

The distinction between primary and secondary urethral cancers is crucial, as they necessitate different management approaches. Primary urethral carcinoma is defined as carcinoma diagnosed initially in the urethra, whereas secondary urethral carcinoma represents the recurrence or spread of malignancy originating from other parts of the urinary tract, most commonly bladder or prostate cancer [2,3].

The etiology of primary urethral cancer is multifactorial and exhibits sex-specific differences. In females, risk factors include recurrent urinary tract infections [2,4], urethral diverticula [2,5], chronic irritation, human papillomavirus type 16 and 18 infections [4], sexual activity, and parturition [6]. In males, risk factors include urethral strictures [2,4], chronic irritation subsequent to intermittent catheterization [4], urethroplasty [2,7], EBRT [2,7], brachytherapy [2,8], chronic urethritis or inflammation of the urethra due to sexually transmitted diseases [6] (e.g., condyloma [2,4]), trauma [6], human papillomavirus (HPV) type 16 [9], and lichen sclerosus [2,10]. Notably, the prevalence of HPV is much lower in the male urethra than in the penis [4].

Only one European study, published over two decades ago (data from 1995 to 2002), has reported on PUC incidence, indicating significantly lower incidence in Eastern Europe (~0.3 per million) compared to other regions (0.9–1.3 per million). There are no studies available on PUC mortality in Europe. The primary objective of this research to estimate and compare the incidence of primary urethral cancer in Europe using cancer registry data during the period 2000–2022, with a particular focus on Eastern Europe and Poland. Furthermore, for illustrative purposes, in this work, available cancer mortality data for primary urethral cancer have been used.

## 2. Materials and Methods

### 2.1. Study Population

Our study is based on country-level data and is descriptive in nature. For case identification, we used ICD-10 code C68.0, which specifically refers to malignant neoplasm of the urethra, excluding other unspecified urinary organ malignancies classified under codes such as C57.9 (female genitourinary NOS (not otherwise specified)) and C63.9 (male genitourinary NOS). We selected only those countries providing data specifically for C68.0 to ensure precise targeting of primary urethral cancer cases.

The registers from which the data were obtained encode this location in accordance with ENCR guidelines. As a result, the incidence data only include actual new primary cancers.

### 2.2. Topographic Classification

Cases of PUC are drawn upon request from national cancer registries across Europe.

This is a collaborative study between countries based on a shared protocol. We have collected age-standardized incidence rate (Europe) for age groups 0+ and 40+ for the years 2000–2022 and incident cases in the last observed year in 5-year age groups.

### 2.3. Selection Criteria

We analyzed incidence data from 11 registers (Austria, Belgium, Bulgaria, Lithuania, Latvia, Germany, Slovenia, Switzerland, Hungary, Poland, and Czech Republic). Most (7 out of 11) registry databases categorized PUC under the general classification (C68—malignant neoplasm of other and unspecified urinary organs); consequently, data from these registries were excluded.

Cases with a diagnosis of ICD-0-3 C68.0 are aged over 18, diagnosed up to 2022 and followed up for vital status ascertainment to the end of 2023.

The Belgian registry does not share data in 5-year age groups, providing grouped data by age only for the age groups 0+, 0–39, and 40+; thus, it was also excluded from the analysis.

### 2.4. Statistical Analysis

**Figure 1:**
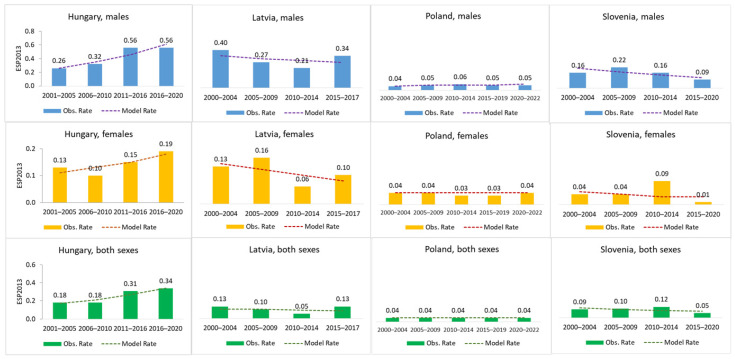
PUC incidence in 4 European Countries (2000–2009 and 2010–2019).

Incidence is presented as age-standardized incidence rates, calculated according to the European Standard Population 2013 (ESP2013) (Figure 1). Due to the rarity of PUC (and consequently the extremely low incidence rates), the authors chose to visualize the data using bar charts representing following calendar time periods: for Hungary (2001–2005; 2006–2010; 2011–2016; and 2016–2020), for Latvia (2000–2004; 2005–2009; 2010–2014; and 2015–2017), for Poland (2000–2004; 2005–2009; 2010–2014; 2015–2019; and 2020–2022), and for Slovenia (200–2004; 2005–2009; 2010–2014; and 2015–2020). To assess the dynamics of incidence over time, the Joinpoint Regression Model version 5.2.0.0 was employed to estimate the Annual Percent Change (APC).

To analyze mortality in European countries (27 European countries), we used data from the WHO Mortality Database [11], available from: https://www.who.int/data/data-collection-tools/who-mortality-database (accessed on 22 May 2025); data downloaded as ZIP file). To compare this data, we have developed maps illustrating the mortality rates of primary urethral cancer (PUC) for individuals aged 45 and above. The maps were prepared by the authors of the article using open-source libraries in Python (GeoPandas—The pandas development team. (2020). pandas-dev/pandas: Pandas (Version 1.0.5) [Computer software]. Zenodo. https://doi.org/10.5281/zenodo.3509134, accessed on 22 May 2025). The standardization of the mortality rates allows for the unification of the studied populations in terms of age structure. To achieve this, mortality rates for specific age groups are used and applied to a standard population structure, in this case the European Standard Population (ESP2013) [12].

The gradation used in the maps was based on the Fisher–Jenks method of natural divisions [13], maximizing the variance between classes and minimizing the variance within classes. The maps present rates based on data aggregated for the period 2013–2022.

For Poland, we conducted the analysis additionally based on data from the PNCR covering the years 2000–2022, to analyze incidence and mortality trends, stratified by sex, calculating 5-year moving averages of rates standardized to ESP2013.

## 3. Results

**Figure 2:**
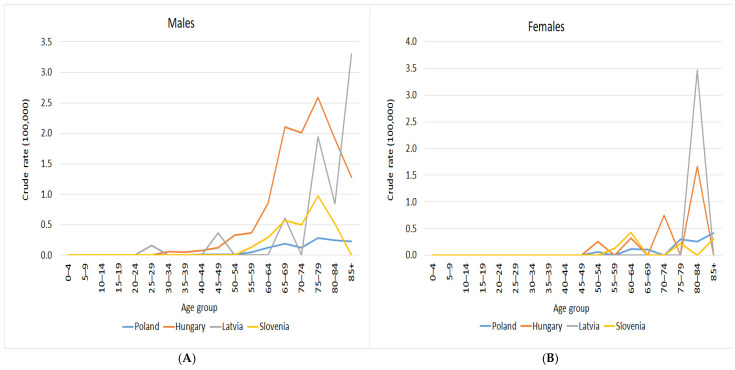
(**A**) Crude incidence rates of PUC collected in 4 cancer registries (2000–2019) by gender and age (males); and (**B**) crude incidence rates of PUC collected in 4 cancer registries (2000–2019) by gender and age (females).

Based on data from 2000 to 2019 for PUC (C68.0) for four countries (Poland (https://onkologia.org.pl/pl, accessed on 22 May 2025), Latvia (https://www.spkc.gov.lv/lv, accessed on 22 May 2025), Slovenia (https://www.onko-i.si/en/crs, accessed on 22 May 2025), and Hungary (https://onkol.hu/nemzeti-rakregiszter-es-biostatisztikai-kozpont, accessed on 22 May 2025), we identified that 94% of incidence occurred in individuals aged over 45 years (Figure 2). Based on data from 2000 to 2022 for PUC for Poland, we also identified that 94% of death occurred in individuals aged over 45 years.

Our analysis included data from four countries: Poland, Latvia, Slovenia, and Hungary (Figure 1).

We performed the analysis on small numbers due to the rare incidence of this cancer. Observed differences between countries could merely reflect variations in how each country records and categorizes cancer cases. These disparities may not accurately represent differences in PUC incidence in Europe.

PUC incidence was the highest in Hungary among both sexes, followed by Latvia. Incidence rates in Slovenia were significantly lower than in Hungary and Latvia. Poland exhibited the lowest PUC incidence rate in both sexes.

In light of the results obtained, only for Hungary is there a statistically significant increase in the incidence rates in men (APC 5.84) (Table 1).

The inherent confidence intervals indicate the lack of significant differences between both analyzed periods in individual countries.

**Figure 3:**
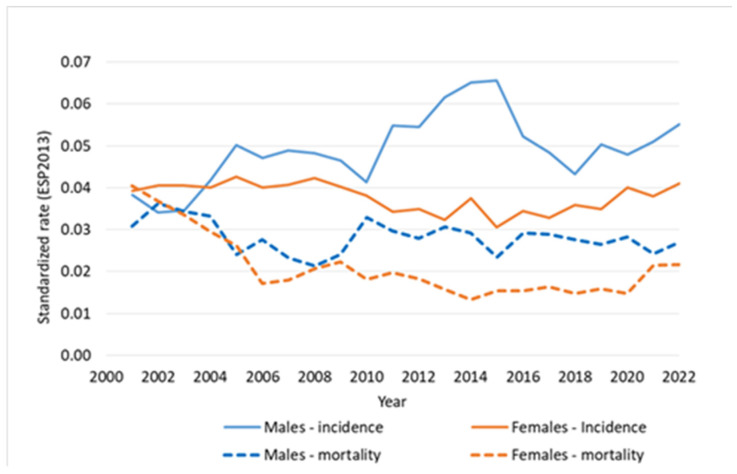
PUC incidence and mortality trends, Poland, 2000–2022.

Fluctuations in rate values due to the small population and the rarity of the disease prompted us to present trends in the form of a line chart for better readability of the data. Since the main target of the analysis is Poland, we presented trends for this country. We used a 5-year moving average. On the Y axis, we presented age-standardized incidence and mortality rates (ASMR), calculated according to the European Standard Population 2013 (ESP2013). Between 2013 and 2022 in Poland, there were 63 new cases among women and 69 among men, indicating a 10% higher incidence in men. The evaluation of incidence trends for PUC in Poland is hindered by interpretative limitations arising from the rarity of this malignancy. Nevertheless, analysis of incidence among males indicates a slight increase beginning in 2018, whereas among females, a similar upward trend appears to have started earlier, around 2014 (Figure 3).

**Figure 4:**
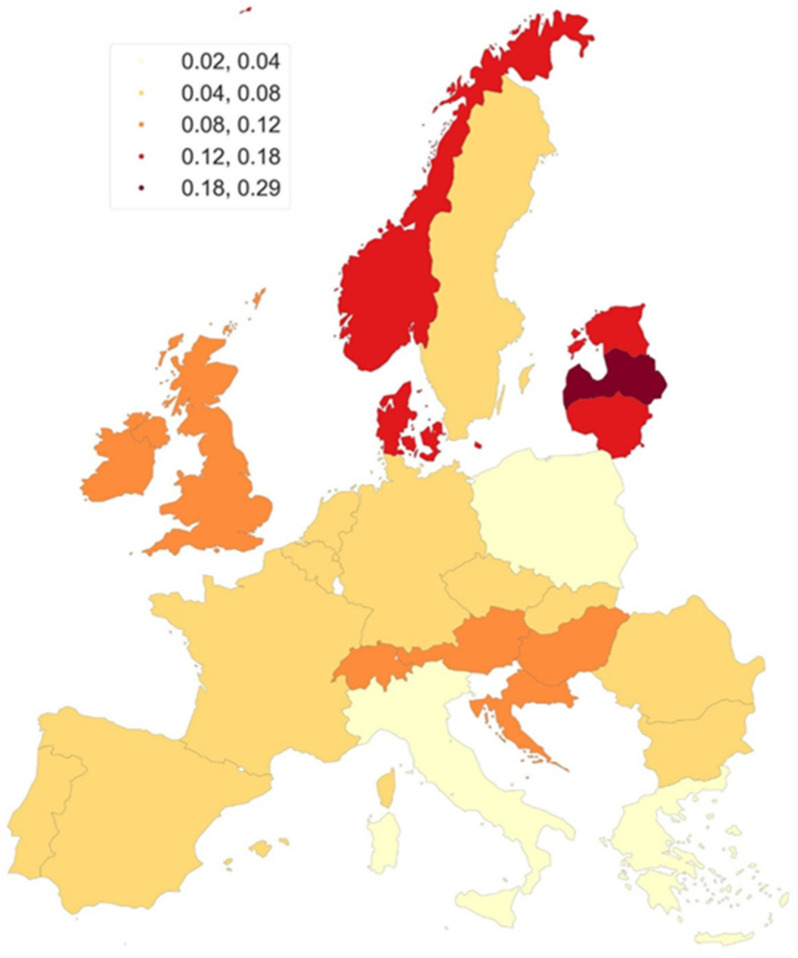
European map of PUC mortality, women aged 45+ (2013–2022), WHO Mortality Database [Internet]. Available from: https://www.who.int/data/data-collection-tools/who-mortality-database, accessed on 22 May 2025.

**Figure 5:**
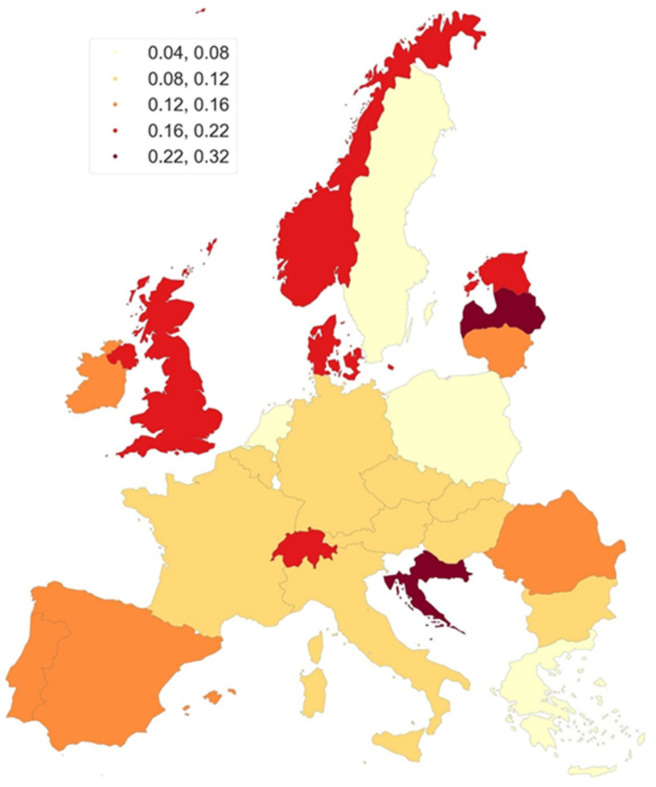
European map of PUC mortality, men aged 45+ (2013–2022), WHO Mortality Database [Internet]. Available from: https://www.who.int/data/data-collection-tools/who-mortality-database, accessed on 22 May 2025 (data downloaded as ZIP file). The maps were prepared by the authors of the article using open-source libraries in Python (GeoPandas—The pandas development team. (2020). pandas-dev/pandas: Pandas (Version 1.0.5) [Computer software]. Zenodo. https://doi.org/10.5281/zenodo.3509134 (accessed on 25 May 2025). The authors of the article grant permission to Springer Nature Limited to publish the images under a CC BY open access license in all formats, i.e., print and digital.

Utilizing the available data, we have developed maps illustrating the mortality rates of primary urethral cancer (PUC) for individuals aged 45 and above. Figure 4 depicts the map for women, while Figure 5 does so for men. Among women aged 45 years and older, Latvia had the highest ASMR (0.18–0.28), with intermediate rates observed in Denmark, Estonia, Lithuania, and Norway (0.12–0.18). Ireland, the United Kingdom, Hungary, Croatia, Austria, and Switzerland were categorized in the second tier of intermediate mortality rates, with ASMRs ranging from 0.04 to 0.08. The third tier, with ASMRs between 0.08 and 0.152, included Sweden, Portugal, Spain, France, Belgium, the Netherlands, Germany, Czechia, Slovakia, Romania, and Bulgaria. Lower ASMRs (0.02–0.04) were found in Italy, Greece, and Poland (Figure 4).

In men aged ≥45 years, Latvia and Croatia had the highest mortality rates (ASMR 0.22–0.32). Intermediate mortality rates were observed in Norway, Denmark, the United Kingdom, Estonia, and Switzerland, with an ASMR between 0.16 and 0.22. Ireland, Lithuania, Portugal, Spain, and Romania exhibited slightly lower intermediate mortality rates, with an ASMR of 0.12 to 0.16. France, Belgium, Germany, Czechia, Slovakia, Austria, Hungary, Italy, and Bulgaria were categorized in the next tier of intermediate mortality rates, with an ASMR of 0.08 to 0.12. The lowest mortality rates, with an ASMR of 0.04 to 0.08, were found in Sweden, the Netherlands, Greece, and Poland.

Latvia exhibited the highest PUC mortality for both sexes, while Poland and Greece consistently showed the lowest mortality rates. Mortality rates were generally higher in men compared to women across Europe.

During 2013–2022, Poland registered 28 female and 35 male deaths from PUC, reflecting a 25% higher mortality rate among men, consistent with the European pattern. Although PUC mortality is relatively low, determining the trend trajectory is challenging due to annual fluctuations in mortality rates. Despite these fluctuations, the overall mortality rate has remained constant (Figure 3).

## 4. Discussion

Our results confirm that PUC incidence is consistently higher in men than in women across the examined European countries, with Hungary showing the highest incidence and Poland the lowest.

One of the risk factors for PUC is HPV 16, a highly oncogenic type. A hrHPV prevalence of 11.15% (9.73% age-representatively) was found in Hungary, similar to the world average, but higher than the European average [14], which may be one of the reasons for the high PUC incidence in Hungary.

The lowest PUC incidence in Poland can be evidence of proper reporting of PUC cases. Poland is one of the few European countries providing data specifically for C68.0 and provides this data for every year from 2000 to 2022.

PUC mortality predominantly affects individuals over 45 years of age. Among women, Latvia had the highest mortality rates, while Italy, Greece, and Poland reported the lowest. In men aged ≥45, the highest mortality was in Latvia and Croatia, while Sweden, the Netherlands, Greece, and Poland show the lowest rates.

There are no articles on the epidemiology of primary urethral cancer in Latvia, while Latvia has one of the highest cancer death rates in the European Union. According to EUROSTAT data [15], in 2021 the standardized cancer mortality rate in Latvia was ~283.5 deaths per 100,000 people—higher than the EU average. Significant gaps have been identified in prevention, early detection, and access to cancer care. The share of the population that participates in screening programs is also low [16].

In Poland, the lowest PUC mortality was observed in both sexes. Due to the rarity of the cancer and the small numbers involved, it is difficult to justify the reasons for this phenomenon.

Historical reports from the 19th century by Boiven & Deuges (1833) and Thiaudierre (1834) first described urethral cancer, but in the 1980s, PUC was more often diagnosed in women than men across all countries [3]; our study highlights a reversal of this pattern in recent decades and underscores the importance of updated epidemiological data to better understand the disease’s current burden and guide clinical and public health strategies.

Due to scarce contemporary literature, comparison is limited. A prior European study using RARECARE data from 1995 to 2002 and encompassing 64 cancer registries reported higher incidence in elderly patients (≥75 years) and a male predominance. The five-year relative survival rates differed by histological type: squamous cell carcinoma (SCC) 69%, transitional cell carcinoma (TCC) 52%, and adenocarcinoma (AC) 48% [4].

The remaining studies on primary urethral cancer (PUC) incidence, cancer-specific mortality, and the stage of primary urethral cancer are based on the US SEER registry (Surveillance, Epidemiology, and End Results) [6,7,8,9]. The authors of studies based on the SEER registry agree that the incidence of primary urethral cancer is highest in males, elderly patients, and African Americans [6,17,18]. Moreover, according to SEER data, female patients with urethral cancer present with a higher disease stage [17] and exhibit higher cancer-specific mortality [17]. However, SEER data are influenced by the US insurance system, limiting direct comparisons with European populations that benefit from universal health coverage.

In the context of primary urethral cancer (PUC), prognostic factors for men include tumor location and stage; for women, tumor size is also significant [6]. Diagnosis often occurs at an advanced stage with locoregional spread [6]. Furthermore, distant metastasis is reported in approximately 6% of patients [6]. Surgical intervention and radiation therapy (RT) are advantageous in the early stages of the disease [6]. In contrast, advanced-stage disease necessitates a multimodal treatment approach, including induction chemotherapy followed by surgery or RT, and concurrent chemoradiation with or without surgery [6]. Regional lymph node involvement is observed in one-third of patients, irrespective of sex [6]. Treatment at academic centers involves more frequent use of neoadjuvant/adjuvant chemotherapy and radiation compared to nonacademic centers. They perform radical surgery in 34.1% of patients, in contrast to 14.5% in community programs, and regional lymphadenectomy in 31.6% of patients compared to 13.2% [19]. Academic centers also exhibit superior overall survival rates for PUC in pairwise comparisons and are associated with a decreased risk of death, with a hazard ratio of 0.858 (95% confidence interval 0.749−0.983) [19].

Centralization of care to high-volume centers improved outcomes in bladder, prostate, penile, testicular, and renal cancer [15]. There are not many publications regarding the effect of centralization of care for low-incidence urologic cancers [20]. We found only one publication devoted to this topic in PUC [19]. Authors from the US have analyzed 6476 patients diagnosed with PUC in centers reporting to the National Cancer Database from 2004 to 2016. They identified a significant difference in overall survival based upon the type of treatment facility. Five-year overall survival estimates were 35% for community, 38% for comprehensive community, 43% for academic, and 40% for integrated network cancer programs, respectively. Academic centers had superior overall survival and were associated with decreased risk of death in comparison with nonacademic centers. No study has yet been conducted in Europe on centralization in PUC and its impact on mortality. However, PUC is a rare cancer, similar to penile cancer, and centralized care for penile cancer has been introduced in several countries. In England, the penile cancer mortality decrease was particularly evident for men born after 1915 and remained relatively stable for the more recent cohorts of men, indicating a genuine improvement in treatment efficacy in recent years in this country, which can be attributed to centralization [21]. Penile cancer centralized care was also introduced in Netherlands and Scandinavia (Denmark, 2009), and is discussed in Germany and Austria, and in Poland, the first Penile Disease Outpatient Clinic was opened in early 2020 at the Invasive Medicine Center of the University Clinical Center in Gdańsk [22]. We believe that in the case of PUC, the centralization of health care is also a positive development that should be promoted.

PUC staging is not standardized and varies across centers [23]. It is worth noting that MRI is the most sensitive imaging technique for evaluating tumor staging and local invasion in PUC patients [24]. In accordance with EUA, AFU, and NCCN guidelines, MRI should be systematically performed for PUC diagnosis [2,25,26].

Regarding recurrences, the proportion of non-metastatic patients that experienced a recurrence event ranges between 53% [27] and 65% [23]. The places most frequently affected by metastases are the lungs and distal lymph nodes [23,25].

In advanced-stage disease, association of treatments in a multimodal strategy reduces recurrences and increases overall survival and disease-free survival [28,29,30,31,32].

## 5. Study Strengths and Limitations

This study presents the first contemporary, registry-based comparison of primary urethral cancer (PUC) incidence and mortality across European countries, filling a significant gap in the epidemiological literature. Its major strength lies in the use of data from official national cancer registries and the WHO Mortality Database, which ensures high reliability and cross-national comparability. The analysis focuses specifically on ICD-10 code C68.0, enabling targeted assessment of urethral cancer distinct from other urogenital malignancies. Additionally, the stratification of data by sex and age group provides clinically relevant insights and supports targeted public health strategies.

However, the study also has several limitations. The rarity of PUC inherently limits statistical power and the ability to assess long-term trends with confidence.

Even with a five-year moving average, age-standardized incidence and mortality rates in Poland show very large changes from year to year, which made it difficult to interpret trends based on formal statistical testing.

Incidence data were available from only four European countries, due to heterogeneous reporting practices and aggregation of C68 codes in many registries. Estimates of the incidence rate are unstable due to the low number of cases, which is in turn caused by the rarity of the disease. Moreover, potential coding inconsistencies and underreporting may affect the accuracy of national datasets and number of cases. Differences in the time frames of available data across countries may also reduce comparability. Finally, the absence of clinical and histopathological details, such as stage and tumor subtype, precludes the further analysis of survival outcomes or treatment patterns.

## 6. Conclusions

Primary urethral cancer is an exceptionally rare urogenital malignancy with limited epidemiological data in Europe, especially concerning site-specific coding (C68.0). This scarcity hampers robust international comparisons and long-term trend analysis.

PUC incidence is consistently higher in men than in women across studied countries, likely reflecting anatomical and biological factors. Though mortality differences exist between countries, absolute case numbers remain low, which limits statistical significance.

Based on the knowledge drawn from the literature presented in the article on the impact of centralization on the increase in overall survival and the decrease in mortality in rare cancers, the authors believe that the centralization of care can improve PUC patient outcomes.

## Figures and Tables

**Table 1:** APC for PUC incidence in 4 European countries (2000–2009 and 2010–2019).

Sex	Country	Year	APC	Lower CI	Upper CI	*p*-Value
M	HUN	2001–2020	**5.84**	**2.30**	**9.48**	**<0.000001**
M	LAT	2000–2017	−1.52	−9.99	7.73	0.681
M	POL	2000–2022	1.62	−0.60	3.80	0.156
M	SLO	2000–2020	−4.05	−14.06	7.23	0.369
F	HUN	2001–2020	3.03	−0.73	6.88	0.162
F	LAT	2000–2017	−3.46	−10.45	3.99	0.419
F	POL	2000–2022	−0.42	−2.10	1.26	0.543
F	SLO	2000–2020	−4.50	−19.60	13.27	0.585
MF	HUN	2001–2020	**4.81**	**1.61**	**8.12**	**<0.000001**
MF	LAT	2000–2017	−1.33	−9.32	7.20	0.735
MF	POL	2000–2022	0.46	−0.72	1.65	0.377
MF	SLO	2000–2020	−3.19	−13.38	8.08	0.466

## Data Availability

Data available on request from the authors.

## Abbreviations

AC—Adenocarcinoma; ASMR—Age-Standardized Mortality Rate; CI—Confidence Interval; CR—Cancer Registry; EBRT—External Beam Radiotherapy; ESP2013—European Standard Population 2013; HPV—Human Papillomavirus; ICD-10—International Classification of Diseases, 10th Revision; PUC—Primary Urethral Cancer; RSR—Relative Survival Rate; RT—Radiotherapy; SCC—Squamous Cell Carcinoma; SEER—Surveillance, Epidemiology, and End Results Program; TCC—Transitional Cell Carcinoma; WHO—World Health Organization.
